# Selective agonists of the glucocorticoid receptor (SEGRA) as an alternative to glucocorticoids: a pilot study of their effects on normal mouse brain tissue in the context of an acute peripheral inflammatory model *in vivo*

**DOI:** 10.3389/fonc.2026.1778726

**Published:** 2026-07-13

**Authors:** Stanislav D. Aladev, Maxim O. Politko, Dmitry K. Sokolov, Galina M. Kazanskaya, Svetlana V. Aidagulova, Elvira V. Grigorieva

**Affiliations:** 1 Institute of Molecular Biology and Biophysics Federal Research Center of Fundamental and Translational Medicine (FRC FTM), Novosibirsk, Russia; 2 Laboratory of Cellular Biology, Novosibirsk State Medical University, Novosibirsk, Russia; 3 Karolinska Institutet, Stockholm, Sweden

**Keywords:** brain extracellular matrix, dexamethasone, glial fibrillary acidic protein, glucocorticoid, glycosaminoglycan, heparan sulfate, proteoglycan, selective glucocorticoid receptor agonist

## Abstract

**Background:**

Glucocorticoids (GCs) are used to treat multiple pathologies; however, their prolonged use leads to numerous adverse effects. Non-steroidal selective agonists of the glucocorticoid receptor (SEGRAs) have the potential to be a worthy substitute for dexamethasone (DXM). The aim of this study was to compare the effects of SEGRAs and DXM on the cell composition and extracellular matrix of normal brain tissue, with a focus on the acute short-term effects of these drugs.

**Methods:**

C57Bl/6 mice (n = 28) received an intraperitoneal injection of SEGRA (CpdA or CpdA-03) or DXM after carrageenan-induced paw edema. After 7.5 h, brain tissue was analyzed for neurons and astrocytes by IHC and Western blotting (anti-NF and anti-GFAP, respectively). Expression of proteoglycan (PG) core proteins and heparan sulfate (HS) metabolism-involved genes was determined by RT-PCR. The glycosaminoglycan (GAG) content was determined by dot blot and Alcian blue staining.

**Results:**

All studied drugs possessed similar anti-edema activity but differed in their effects on brain tissue. Unlike DXM, SEGRAs had no effect on neurofilament (NF) content in the mouse brain. Regarding astrocytes, although all drugs increased astrocyte number and outgrowth, only DXM and CpdA, but not CpdA-03, induced the appearance of a minor (44kDa) GFAP isoform associated with neurocognitive impairment. Neither DXM nor CpdA-03 affected the expression of genes coding PG core proteins and HS biosynthetic enzymes, and only CpdA upregulated the expression of syndecan-3, neurocan, aggrecan, and biglycan (2.5- to 3.5-fold). At the same time, all drugs decreased the content of total (1.5- to 2.8-fold) and sulfated (2- to 3-fold) GAGs, with the least effect observed for CpdA-03.

**Conclusion:**

Between the two studied SEGRAs, CpdA shows more pronounced effects on the cellular and extracellular components of normal mouse brain tissue compared with DXM. CpdA-03 demonstrated the least effect on brain tissue and may be promising for further development as a less toxic replacement for glucocorticoids.

## 1 Introduction

Glucocorticoids (GCs) are anti-tumor, anti-inflammatory, and anti-allergic drugs that are used in the treatment of various diseases, such as glioblastoma (GBM), COVID-19, and others (1, 2). However, long-term GC use has numerous adverse effects, including severe immunosuppression and metabolic changes, which may impair the survival of GBM patients (3, 4). Because of this, controversial aspects of GC use and the need to improve anti-edema therapy are currently being actively discussed (5, 6).

One potential approach could be the development of a less toxic replacement for GCs, and selective glucocorticoid receptor agonists (SEGRAs) may be considered. Several non-steroidal SEGRAs have been developed and have entered clinical trials as anti-inflammatory glucocorticoid receptor (GR) ligands with reduced side effects. One such GR modulator is the molecule 2-(4-acetoxyphenyl)-2-chloro-N-methylethylammonium chloride, Compound A (CpdA), which was first isolated from the traditional African plant *Sutherlandia frutescens* (7). It is known that GR is able to regulate gene expression in the cell nucleus by a transactivation mechanism via GR homodimer binding to GC-responsive elements, or a transprepression mechanism, in which GR interacts with various transcription factors, such as NF-kB (8, 9). It has been shown that CpdA acts as a dissociated GR ligand, strongly competing with GC for GR binding, poorly inducing GR-mediated gene activation, but effectively inducing GR transrepression. In the review by Rogliani et al., CpdA was described as an inhibitor of airway inflammation via a classical mechanism without inducing the transactivation mechanism, and the overall anti-inflammatory effect was GR-dependent through inhibition of NF-kB activity in a mouse model of asthma (10). CpdA administration increased GR binding levels by 2-fold, but had no effect on GR protein content or GR mRNA levels in a BWTG3 mouse hepatoma cell culture (11). CpdA is as effective as other GCs *in vivo*, but it has an improved therapeutic index and fewer side effects (12).

The functional effects of SEGRAs remain poorly understood. CpdA administration to C57Bl/6 mice significantly suppressed the clinical symptoms of experimental autoimmune encephalomyelitis (EAE), and the therapeutic effect of CpdA occurred in the absence of hyperinsulinemia, i.e., without side effects, unlike dexamethasone (DXM) (13). Multiple administrations of a cyclodextrin-encapsulated CpdA compound to inbred female rats significantly prolonged the survival time of corneal grafts in an experimental keratoplasty model (14). One of the developed SEGRAs, mapracorate, has already shown its effectiveness in clinical studies by inhibiting the production of proinflammatory mediators in LPS (lipopolysaccharide)-stimulated untreated macrophages Raw 264.7 (15). Systemic CpdA administration did not cause hearing threshold shifts, but intratympanic CpdA injection resulted in persistent hearing loss by 2- to 4-fold in a guinea pig hearing loss model (16).

CpdA also demonstrates certain anti-tumor effects. CpdA inhibits lymphoblastic leukemia growth in primary cell cultures (17); the growth and viability of PC3 prostate cancer xenografts and Granta-519 mantle cell lymphoma xenografts *in vivo* (18); and the growth and proliferation of MDA-MB-231 and MCF7 breast cancer cell lines without promoting drug resistance or attenuating the cytotoxicity of leading therapeutic agents (19). Administration of CpdA and the experimental SEGRA drug ZK 216348 inhibited intestinal epithelial cell proliferation in an *in vitro* intestinal wound model (20). To our knowledge, these are the main published results related to the functional effects of CpdA.

Thus, CpdA might be a potential candidate to substitute steroidal GCs; however, its low solubility and instability in aqueous solutions pose an objective challenge for its development as a therapeutic drug. To overcome these difficulties, several CpdA analogs with better solubility and stability in aqueous solutions were synthesized and designated as CpdA-01–CpdA-08 (21). Among these SEGRAs, CpdA-01 demonstrated the highest anti-proliferative activity on Granta cells in an *in vitro* hemoblastosis experimental model, whereas CpdA-03 demonstrated the strongest tumor growth inhibition after P388 mouse lymphoma implantation into female DBA/2 mice *in vivo* (21). In addition, CpdA-03 also demonstrated the highest antiproliferative activity in luminal MCF-7 and triple-negative breast MDA-MB-231 cancer cell lines and induced accumulation of cells in the G1 phase of the cell cycle (22).

In terms of SEGRA effects on brain tumors or normal brain tissue, only one relevant study was found (23). This study demonstrated that the SEGRA drug AL-438 does not affect proteoglycan (PG) aggrecan biosynthesis and gene expression, unlike DXM administration, which significantly decreases aggrecan biosynthesis in a chondrocyte cell culture derived from fetal mouse metatarsals (23).

At the same time, it was shown that DXM is able to change the structure and composition of glycosylated components (PGs, glycosaminoglycans [GAGs]) in the normal brain extracellular matrix (ECM). DXM treatment affects both PG core protein expression and heparan sulfate (HS)/chondroitin sulfate (CS) content in rat brain in a dose-dependent manner (24). These changes are more pronounced upon combined treatment with a DXM–temozolomide combination, which results in the accelerated adhesion, proliferation, and invasion of glioblastoma (GB) cells into brain organotypic slices *ex vivo* and more active growth and invasion of experimental xenograft GB tumors into the brains of SCID mice in an experimental model of GB relapse *in vivo* (25). Moreover, GB xenografts grown in DXM pretreated animals demonstrate attenuated HS biosynthesis and decreased HS content (26). A detailed study of DXM effects on brain ECM in short term (1–10 days) and long-term (15–90 days) experiments supports its ability to affect glycosylated components of brain ECM (27, 28).

In addition to affecting the matrix, DXM can also modify cell composition of normal mouse brain tissue by decreasing the number of microglial cells and increasing the number of astrocytes. Long-term use of DXM results in the inhibition of myelin formation and the appearance of truncated glial fibrillary acidic protein (GFAP) isoforms, suggesting its ability to contribute to neurodegeneration-like changes in normal mouse brain tissue (29). Thus, DXM can affect normal brain tissue, which may contribute to the development of long-term neurological side effects.

When studying SEGRAs as a potential replacement for glucocorticoids, it is important to investigate whether they affect brain tissue in a manner similar to DXM. This study aims to perform a comparative analysis of the effects of CpdA, CpdA-03, and DXM on cell composition (astrocytes, neurofilaments) and components of the extracellular matrix (PGs and glycosaminoglycans [GAGs]) of normal brain tissue in a mouse paw edema experimental model.

## 2 Materials and methods

### 2.1 Animals

Male C57Bl/6 mice, 12 weeks old and weighing 22–30 g, were used for this study. The animals (n = 28) were obtained from the SPF facility of the Institute of Cytology and Genetics (Novosibirsk, Russia) and kept in polycarbonate cages with free access to food and water. The cages were placed in a ventilated room with a 12/12-h light/dark cycle, a temperature of 25 ± 1 °C, and 50%–60% humidity.

### 2.2 Carrageenan-induced edema experimental model

Mice were injected with 50μL 1% carrageenan (22049, Sigma-Aldrich, St. Louis, MO, USA) subcutaneously into the hind left paw. The height and width of the middle of the paw were measured with a caliper every hour. After 1 h, the mice were randomly assigned to experimental groups and received a 200 μL intraperitoneal injection of saline (control group, n = 7), CpdA (Sigma, USA) or CpdA-03 (a generous gift from Dr. Ekaterina Lesovaya, N.N. Blokhin National Medical Research Center of Oncology, Moscow, Russia) (both 7.5 mg/kg), or DXM (KRKA Pharma, Novo Mesto, Slovenia) (1 mg/kg) (three experimental groups, n = 21). These doses correspond to those used in (21) and were justified in previous studies for SEGRAs (18, 30) and DXM (27, 28). After 7.5 h (when both early phase (0–1 h) and late phase (1–6 hours) had been completed), the mice were sacrificed by decapitation using a guillotine according to animal euthanasia guidelines (National Research Council Committee for the Update of the Guide for the Care and Use of Laboratory Animals, 2011). The brain from each animal was collected; one hemisphere was placed in RNAlater solution (Invitrogen; ThermoFisher Scientific, Waltham, MA), and the other was fixed in 10% neutral buffered formalin (BioOptica, Milan, Italy) for paraffin blocks. All subsequent experiments and analyses were performed in a blinded manner. All procedures involving experimental animals were carried out in accordance with the Directive of the Council of the European Community 2010/63/EU and approved by the local committee on biomedical ethics of the Institute of Molecular Biology and Biophysics FRC FTM (approval no. N4/2017; Novosibirsk, Russia).

### 2.3 Real-time RT-PCR

Total RNA was isolated using a QIAzol Lysis Reagent (Qiagen, San Diego, CA). Reverse transcription was performed using the RevertAid H Minus First Strand cDNA Synthesis Kit (ThermoFisher Scientific, Waltham, MA). Real-time PCR was performed on a CFX90 instrument (BioRad, Hercules, CA) using HS-qPCR SYBR Blue (Biolabmix, Novosibirsk, Russia) and primers (28). The expression level was determined using the formula (2^-ΔCt^) × 1000, where ΔCt = Ct (gene)− Ct (Gapdh) for each sample (animal).

### 2.4 Alcian blue staining for total and highly sulfated GAG content

GAG content in the samples was determined by staining paraffin sections with Alcian blue at different pH values (pH = 2.5 for total GAGs and pH = 1.0 for highly sulfated GAGs) (BioVitrum, Saint Petersburg, Russia), according to the manufacturer’s instructions, with the addition of Ehrlich’s hematoxylin. The stained samples were analyzed using an AxioScope A1 microscope with an AxioCam MRc5 camera (Zeiss, Reutlingen, Germany) at 400× magnification. The resulting images were processed for quantitative analysis using the chromoanalytical algorithms of the ZEN Blue 2.6 (Zeiss, Reutlingen, Germany) to determine the ratio of the tissue area showing a specific staining signal to the total image area, expressed as a percentage.

### 2.5 Immunohistochemistry

The paraffin-embedded mouse cerebral hemispheres were used for histological and IHC analyses. Paraffin coronal sections 3–4 μm thick, obtained at a distance of 5 ± 0.5 mm from the bregma, were stained with hematoxylin/eosin (BioVitrum, 05-006, Saint Petersburg, Russia) for 15 min at room temperature or used for immunohistochemical staining for neurofilaments (NF) or GFAP. Indirect immunoperoxidase reaction was performed with primary antibodies against NF (ThermoFisher Scientific, MS-359-R7, Waltham, MA) or GFAP (ThermoFisher Scientific, ASTRO6, Waltham, MA) for 60 min at room temperature. The reaction products were visualized with the Mouse and Rabbit Specific HRP/DAB (ABC) Detection IHC Kit (PrimeBioMed, 78-310004-15, Moscow, Russia).

### 2.6 Western blotting

Mouse brain tissue was homogenized in RIPA buffer (ThermoFisher Scientific, Waltham, MA) supplemented with protease inhibitors Complete Mini (Roche, Mannheim, Germany). After 10% PAAG gel electrophoresis, proteins were transferred to an Immobilon-PVDF membrane (ThermoFisher Scientific, Waltham, MA) and blocked using 5% dry milk (BioRad, Hercules, CA) in PBST buffer for 60 min. The membranes were incubated with antibodies against GFAP (Cloud-Clone Corp., PAA068Mu01, Katy, TX, 1:2000) or Gapdh (Abcam, ab181602, Cambridge, UK, 1:30000) for 60 min, and then with secondary anti-rabbit antibodies (Abcam, ab3578, Cambridge, UK, 1:5000) for 60 min at room temperature. The signal was visualized using Amersham ECL Prime (Cytiva, Marlborough, MA), and detection was performed using BioRad Chemidoc. The obtained data were processed using ImageLab 6.0.1 (BioRad, Hercules, CA).

### 2.7 Dot-blot analysis for HS content

Mouse brain tissue lysates were applied to an Immobilon-PVDF membrane (ThermoFisher Scientific, Waltham, MA) activated in 95% ethanol, blocked in 5% fat-free milk (BioRad, Hercules, CA) in PBST buffer for 60 min, and then incubated with primary anti-HS antibodies (Millipore, MAB2040, Darmstadt, Germany, 1:500), followed by incubation with secondary anti-mouse antibodies (Thermo Fisher Scientific, 150077, Waltham, MA, 1:8000) for 60 min at room temperature. The signal was developed using Amersham ECL Prime (Cytiva, Marlborough, MA) and detected using BioRad Chemidoc (BioRad, Hercules, CA).

### 2.8 Statistical analysis

Statistical analysis of the obtained data was carried out using MS Excel 7.0 (Microsoft, Redmond, WA) and OriginPro 10.1 software (OriginLab, Northampton, MA). Statistical significance was determined using one-way analysis of variance (ANOVA) with Fisher’s *post-hoc* test. The normal distribution was checked in OriginPro 10.1 (OriginLab, Northampton, MA) using the Kolmogorov–Smirnov test. A value of *p* < 0.05 was considered to indicate a statistically significant difference.

## 3 Results

In this study, a comparative analysis of DXM, CpdA, and its most promising derivative, CpdA-03, in terms of their effects on normal brain tissue of experimental mice was performed.

### 3.1 SEGRAs and DXM decrease carrageenan-induced edema in mice

**Figure 1:**
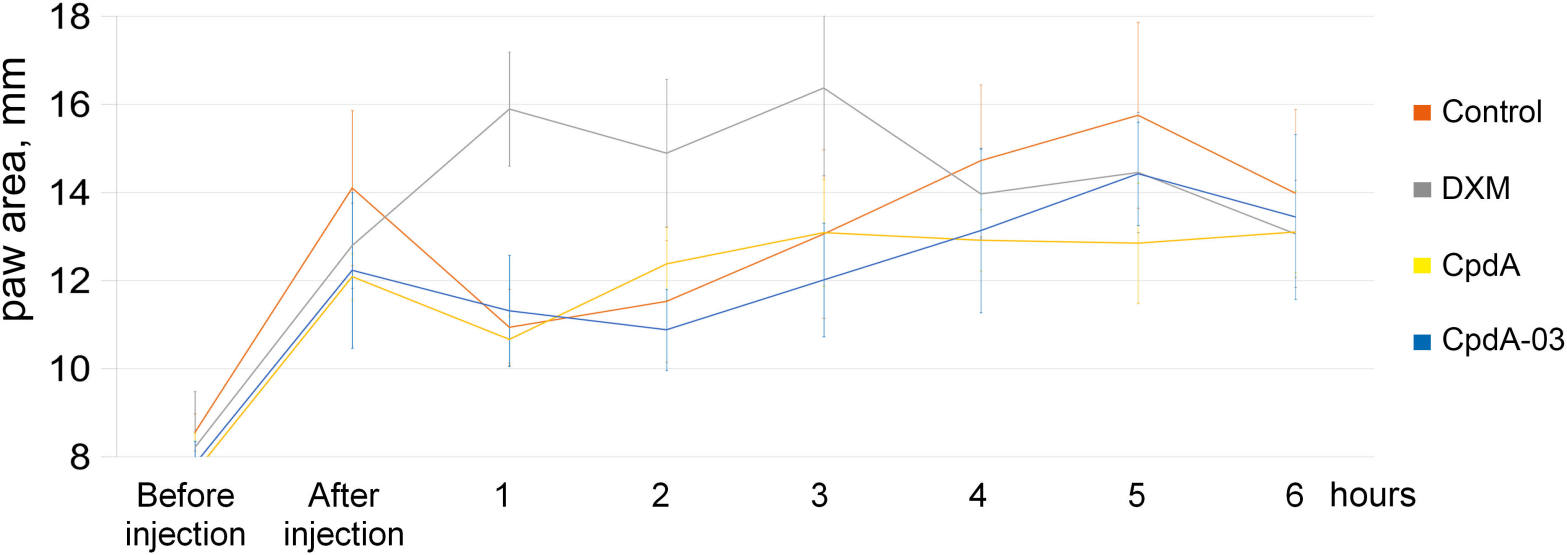
Anti-edema effect of the studied drugs in a carrageenan-induced mouse paw edema model. Paw edema was caused by injection of carrageenan into the hind paw of mice. Control – untreated mice.

The anti-edema activity of the drugs was verified in a carrageenan-induced mouse paw edema experimental model. Carrageenan was injected into the left hind paw of each mouse, and paw size was measured 1 h after the injection, followed by hourly administration of DXM, CpdA, or CpdA-03 (**Figure 1**).

The height and width of the middle of the left hind paw were measured using a caliper before and after 1% carrageenan injection (time point 0) into the left hind paw of the animals. A significant increase in paw size was detected due to the high viscosity of the injected carrageenan (up to 1 h) and the early phase of inflammation (0–1 h). The studied drugs were injected at 1 h, and CpdA/CpdA-03 resulted in a rapid decrease in paw volume, whereas DXM demonstrated a delayed effect. However, over time (by 6 h after carrageenan injection, when the late phase of inflammation had ended), DXM significantly reduced inflammation, while SEGRAs had a weaker effect.

### 3.2 SEGRAs and DXM affect the cell composition of mouse brain tissue

**Figure 2:**
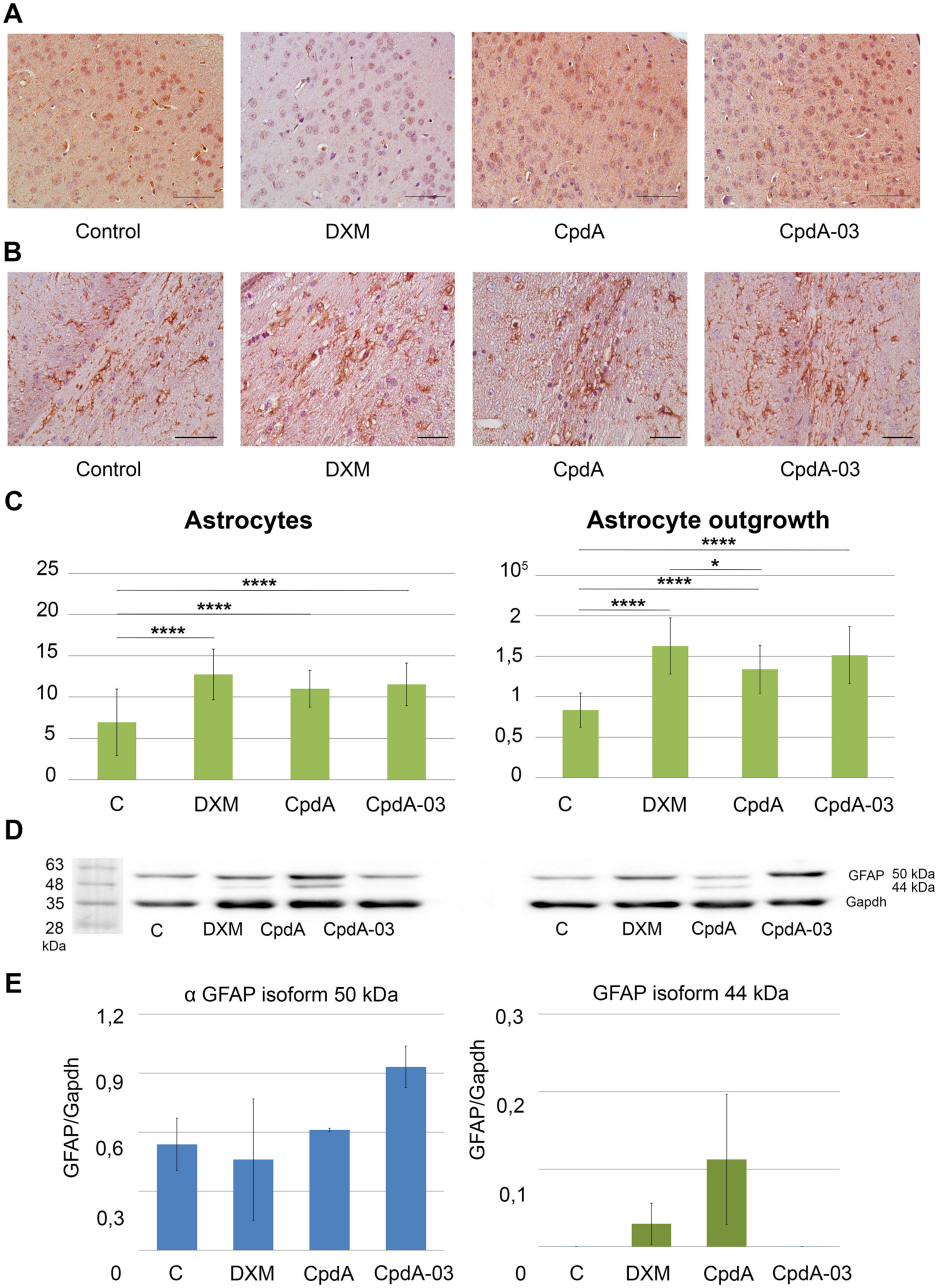
Effects of SEGRAs and DXM on cell composition of the mouse brain tissue. **(A)** Microphotographs of IHC staining with anti-NF antibodies. **(B)** Microphotographs of IHC staining with anti-GFAP antibodies. **(C)** Quantitative analysis of the number of astrocytes and astrocyte outgrowth in the mouse brain, estimated as the stained area of the sample. **(D)** Original microphotographs of Western blotting. **(E)** Quantitative analysis of the GFAP protein α isoform (50 kDa) and the minor GFAP isoform (44 kDa). Control – untreated mice. ANOVA with Fisher’s least significant difference test; *p < 0.05, ****p < 0.0001. Magnification, 400×. Scale bar, 50 μm.

The effects of SEGRAs and DXM on the cell composition (neurons and astrocytes) of the mouse brain were studied by immunohistochemical analysis using antibodies against neurofilament protein (NF) or glial fibrillary acidic protein (GFAP) (**Figures 2A–C**).

According to the IHC staining, only DXM significantly decreased the content of NF protein in mouse brain tissue, whereas CpdA and CpdA-03 did not affect this parameter compared with the control animals (**Figure 2A**). At the same time, all the drugs increased the number of astrocytes (DXM by 1.9-fold [*p* < 0.0001], CpdA by 1.6 fold [*p* < 0.0001], and CpdA-03 by 1.7-fold [*p* < 0.0001]) and astrocyte outgrowth (DXM by 2-fold [*p* < 0.0001], CpdA by 1.7-fold [*p* < 0.0001], and CpdA-03 by 1.9-fold [*p* < 0.0001]) in the mouse brain (**Figures 2B, C**).

To further support these observations, Western blotting with anti-GFAP antibodies was performed (**Figures 2D, E**). Unlike DXM, CpdA and CpdA-03 did not lead to a decrease in the content of the α GFAP isoform (50 kDa); rather, CpdA-03 showed a tendency to increase it. Interestingly, DXM, and to an even greater extent CpdA, led to the appearance of the GFAP minor protein band of 44kDa (**Figures 2D, E**), which has been associated with neurodegenerative changes in animal experimental models (31–33). However, CpdA-03 did not result in the appearance of this 44kDa isoform, supporting interest in this CpdA derivative as a potentially less toxic drug for brain tissue.

### 3.3 CpdA affects the structure of ECM components in mouse brain tissue

**Figure 3:**
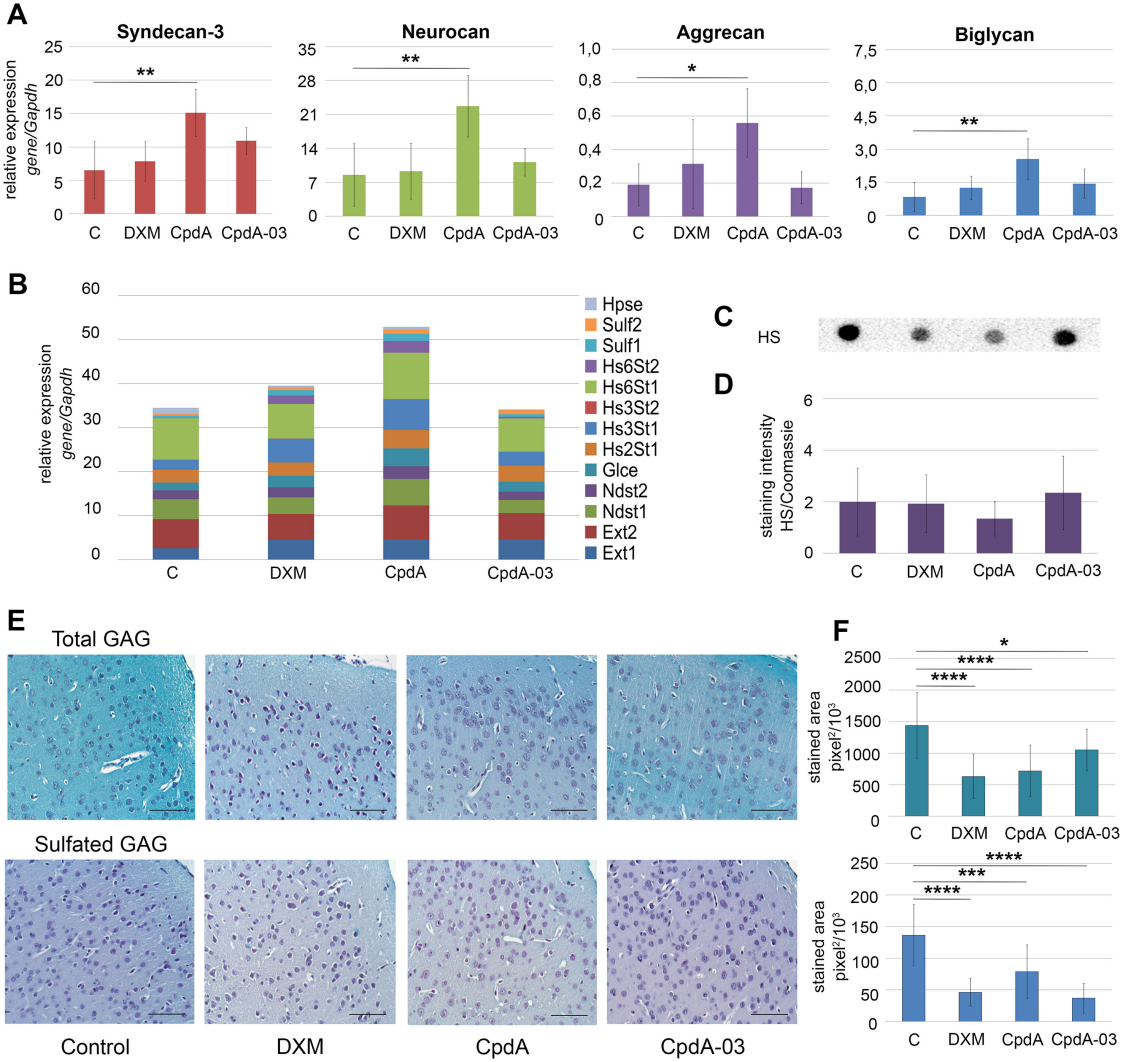
Effects of SEGRAs and DXM on the glycosylated components of mouse brain tissue. **(A)** The mRNA level of the affected PG core proteins. **(B)** Overall transcriptional activity of HS biosynthesis-involved genes. RT-PCR analysis, the intensity of amplified DNA fragments for each gene was normalized to that of Gapdh. **(C)** Original microphotographs of dot blot for HS content using anti-HS antibody. **(D)** Quantitative analysis of HS content. **(E)** Microphotographs of total and sulfated GAGs in mouse brain tissue (stained with Alcian blue at pH = 2.5 and pH = 1.0, respectively). **(F)** Quantitative analysis of total and sulfated GAG content in mouse brain tissue after SEGRAs and DXM administration. Control – untreated mice. ANOVA with Fisher’s least significant difference test; *p < 0.05, **p < 0.01, ***p < 0.001, ****p < 0.0001. Magnification, 400×. Scale bar, 50 μm.

Previously, we demonstrated the ability of DXM to affect composition and content of glycosylated components of mouse brain tissue (PGs, GAGs, and the HS biosynthetic system) in experimental models *in vivo* (24–28). To perform a comparative analysis of the effects of DXM and SEGRAs, expression of PG core proteins and HS biosynthesis-involved genes, together with GAG content, was determined in the experimental groups under investigation (**Figure 3**).

Among the 15 studied PG core proteins (syndecan-1, syndecan-3, glypican-1, perlecan, decorin, biglycan, lumican, brevican, neurocan, aggrecan, versican, Cspg4, Cspg5, CD44, and phosphacan-1), expression of only three genes was upregulated (**Figure 3A**), and this effect was observed only after CpdA administration: perlecan by 3.6-fold (*p* = 0.002), neurocan by 3-fold (*p* = 0.006), and aggrecan by 3-fold (*p* = 0.011) (**Figure 3A**). A similar effect was observed for 13 HS biosynthesis-related genes (*Ext1/2, Ndst1/2, Glce, Hs2st1, Hs3st1/2, Hs6st1/2, Sulf1/2*, and *Hpse*), where only CpdA tended to increase the overall transcriptional activity of the HS biosynthetic system (**Figure 3B**). In fact, unlike CpdA, neither DXM nor CpdA-03 affected the mRNA levels of the majority of the studied genes.

None of the drugs affected HS content in brain tissue (**Figures 3C, D**). At the same time, they decreased GAG content in mouse brain tissue, including both total GAGs (DXM by 2.8-fold [*p* < 0.0001], CpdA by 2.5 fold [*p* < 0.0001], and CpdA-03 by 1.5-fold [*p* = 0.024]) and sulfated GAGs (DXM by 2.8-fold [*p* < 0.0001], CpdA by 2-fold [*p* < 3.86*10^-4^], and CpdA-03 by 3-fold [*p* < 0.0001]) (**Figures 3E, F**). Nevertheless, both SEGRAs performed better than DXM, which reduced GAG content to the greatest extent.

Overall, these results show that SEGRAs had a less significant influence on the cellular composition and glycosylated components of brain tissue than DXM. In turn, among SEGRAs, CpdA-03 appears to be the most promising, as it exerted the least effect on brain tissue.

## 4 Discussion

Comparative analysis of SEGRA and DXM effects on extracellular matrix components (PGs and GAGs) and cell composition of brain tissue (astrocytes and neurofilaments) was performed in this study. We did not find any studies that described the effects of SEGRAs on both extracellular and cell components of brain tissue. Moreover, CpdA-03 is a new experimentally developed drug, and its effects on brain tissue have not been previously reported. Nevertheless, there are studies on the effects of DXM and some SEGRAs on different tissues and cell types, which will be discussed in this section.

In our experimental model, CpdA, and especially CpdA-03, reduced mouse paw edema quickly, whereas DXM initially increased paw size and reduced edema with a delay. There are no studies examining the effects of SEGRAs on mouse paw edema induced by carrageenan, but there are a few reports on the effect of DXM on paw edema. DXM administration at a dose of 1 mg/kg to Swiss mice reduced paw edema induced by 1% carrageenan by 9-fold after 2 h and by 10-fold after 4 h of carrageenan injection in a study of the anti-inflammatory and antinociceptive effects of Serjania marginata leaves (34). In a similar study, DXM administration at a dose of 2.5 mg/kg to Kunming mice reduced paw edema by 4-fold after 4 h of 1% carrageenan injection in a study of the anti-inflammatory effect of the natural phospholipase inhibitor saPLIγ (35). In another study, DXM administration at a dose of 2 mg/kg to Swiss mice reduced cell migration into the peritoneal cavity by 2-fold in a carrageenan-induced peritonitis model in a study of the anti-inflammatory, antinociceptive, and antioxidant activities of Malva branca herb (36). These results demonstrate that the search for a reliable replacement for DXM is ongoing, and attempts are often made to isolate a drug from natural herbs; however, a suitable alternative for reducing paw edema has not yet been identified.

Neither SEGRA affected neurofilaments in mouse brain tissue, whereas DXM administration did cause damage. Our results regarding the effect of DXM on neurofilaments may contribute to research on the influence of GCs on brain tissue cell composition. Moreover, both SEGRA drugs increased GFAP α isoform content, but DXM and CpdA also increased additional GFAP isoform content. These interesting findings are consistent with a study of motor neuron degeneration (Mnd) mice, a mutant line characterized by progressive degeneration of spinal motor neurons. In that study, the 50kDa GFAP isoform, which lacked the head domain, was replaced by other GFAP isoforms with 38kDa and 44kDa molecular masses following DXM administration. This effect was associated with progressive degenerative loss of spinal motor neurons (31). Interesting results were also reported in a study of high-volume training in Wistar rats. The Wistar rats were divided into six groups, and the exhaustion test (ET) was performed. The animals were divided into 6 groups: control, control+ET, moderate-volume (MT) training, MT+ET, high-volume (HV)training, and HV+ET. The contents of the 42 kDa and 39 kDa GFAP isoforms were reduced by 40% and 26%, respectively, in the control + ET and MV-ET groups, whereas the content of the 50 kDa GFAP isoform was reduced by 40%–60%, and the content of the 39 kDa GFAP isoform increased by 7-fold in the HV-ET group. Thus, extensive training in Wistar rats resulted in GFAP isoform profile modification (32). Additional GFAP isoforms were also detected in spinal cords of patients with amyotrophic lateral sclerosis, with a decrease in the content of the GFAP 50 kDa isoform and an increase in the content of the GFAP 44 kDa and 38 kDa isoforms (33). All these data, including our previous results on the capacity of DXM to induce the 44 kDa GFAP isoform in mouse brain tissue (29), demonstrate that among the SEGRAs tested, CpdA but not CpdA-03 is able to modify GFAP isoform profile in mouse brain tissue, similar to DXM.

Furthermore, CpdA injection significantly increased expression levels of some PG core proteins in brain tissue and significantly decreased total and highly-sulfated GAGs in mouse brain tissue. Unfortunately, there are no studies on the effects of SEGRAs on extracellular matrix components of brain tissue, but there are studies investigating the effects of CpdA in other tissues of experimental animals. CpdA administration at a dose of 150 μg contributed to the improvement of clinical symptoms by day 25 after injection in a mouse model of experimental autoimmune encephalomyelitis (13). CpdA can inhibit inflammatory diseases of the bronchi through a transrepression mechanism in a mouse model of asthma (10). CpdA administration five times daily during 35 days to inbred female rats prolonged better survival time of corneal grafts by 4-fold in a rat model of experimental corneal transplantation (14). Moreover, CpdA administration at concentrations of 10 nM–10 μM demonstrated an anti-tumor effect in human lymphoblastic leukemia primary cells by 40%–60% (17). Administration of 8–10 mg/kg inhibited the growth of prostate cancer cell xenografts by 40% and Granta-519 lymphoma cell xenografts by 70% *in vivo* (18). Interestingly, CpdA and another SEGRA drug, ZK 216348, at concentrations of 1–10 μM, also decreased intestinal epithelial cell proliferation by 1.3-fold in an *in vitro* intestinal wound model after 24 h of incubation (20). Despite the positive effects of CpdA, it still has side effects, although these are clearly less pronounced than those associated with steroidal GCs. CpdA injection at dosages of 1 and 10 nM contributed to causing hearing loss in guinea pigs by 2-4-fold between days 3 and 28 after injection (16). Thus, the CpdA compound remains a promising substitute for steroidal GCs, although it is not completely free of adverse effects. Discovering the effects of new synthesized CpdA compounds may facilitate the development of improved treatments for a variety of diseases. The new SEGRA drug CpdA-03, administered at a dose of 7.5 mg/kg, inhibited tumor growth by 78% on day 12 after P388 mouse lymphoma implantation in 5-week-old female DBA/2 mice (21).

Thus, developing successful SEGRA drugs with an improved benefit/risk ratio remains a challenge, as does understanding the extent to which the presence of GR isoforms modulates the biological response to different SEGRAs. Nevertheless, another review reported that CpdA reduced TNF-α-induced NF-kB activation by attenuating IKK phosphorylation and mitogen-activated protein kinase (MAPK) activation. This finding was in stark contrast to DXM, which was unable to affect such relevant pathways (37). Thus, once again, a SEGRA was shown to be superior to classic steroidal GCs such as DXM.

Thus, this study provides the first pilot data on the effects of CpdA and CpdA-03 on mouse brain tissue. However, certain limitations should be acknowledged. In this study, we used only male C57Bl/6 mice because the administration of hormonal drugs such as DXM can affect the sexual cycle of female mice. In addition, only one GC, namely, DXM, was used. In this investigation, we focused on the effects of SEGRAs on the cellular and extracellular components of normal mouse brain tissue compared with DXM. Mice received a single intraperitoneal injection of SEGRAs (CpdA or CpdA-03) or DXM after acute carrageenan-induced paw edema and were euthanized 7.5 h after carrageenan injection. However, tissue-specific pharmacokinetics of CpdA and CpdA-03 in the central nervous system have not yet been studied.

Taken together, these results support further investigations of SEGRAs as potentially less toxic replacements for glucocorticoids.

## Data Availability

The original contributions presented in the study are included in the article/**Supplementary Material**. Further inquiries can be directed to the corresponding author.
