# Supplementary figures and images for: Selective agonists of the glucocorticoid receptor (SEGRA) as an alternative to glucocorticoids: a pilot study of their effects on normal mouse brain tissue in the context of an acute peripheral inflammatory model in vivo
Source: Front Oncol. 2026 Jul 13;16:1778726. doi: 10.3389/fonc.2026.1778726 (PMC13430454; doi:10.3389/fonc.2026.1778726)

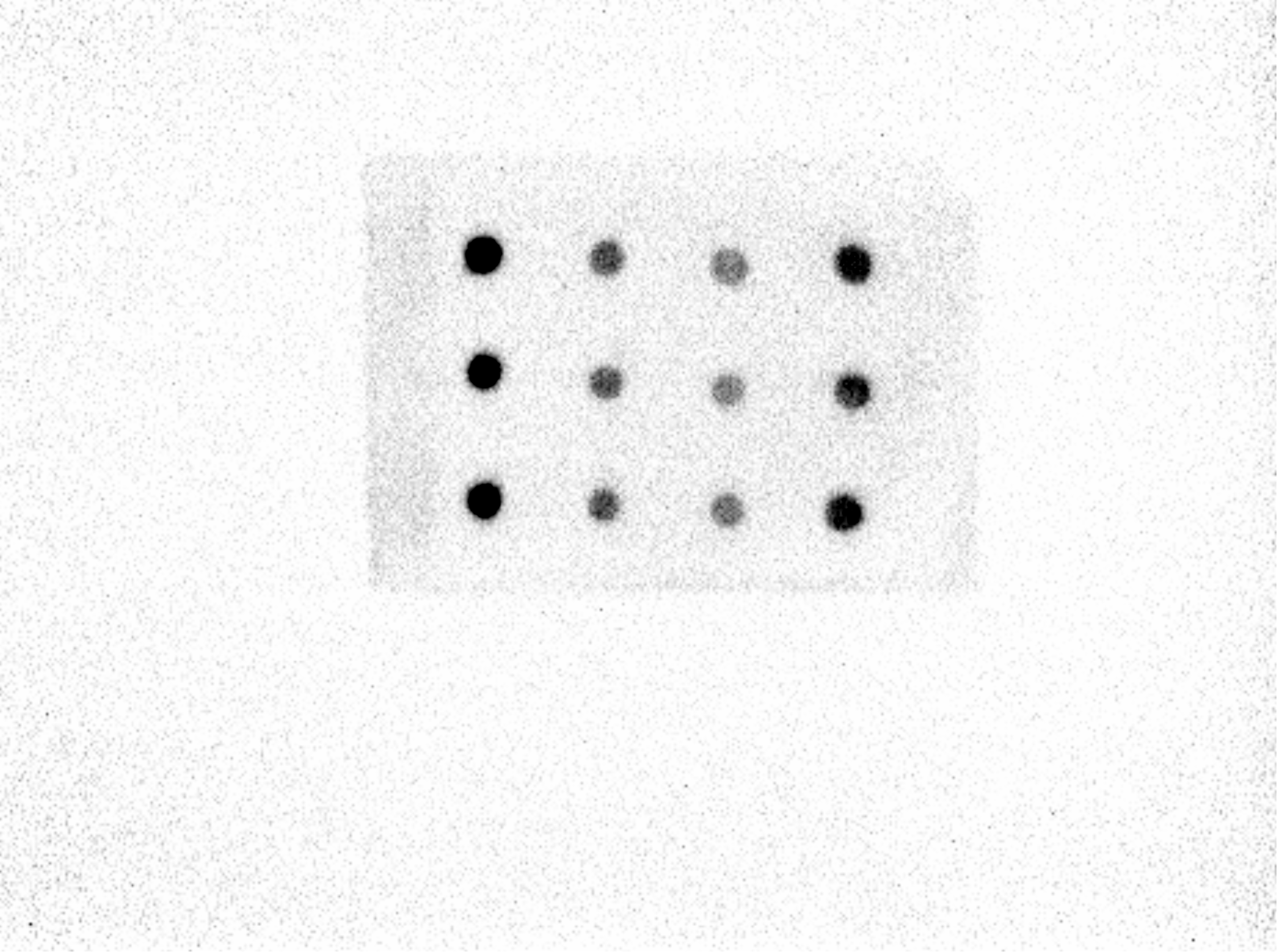

Supplement: Supplementary file 1 [file Image1.tif]

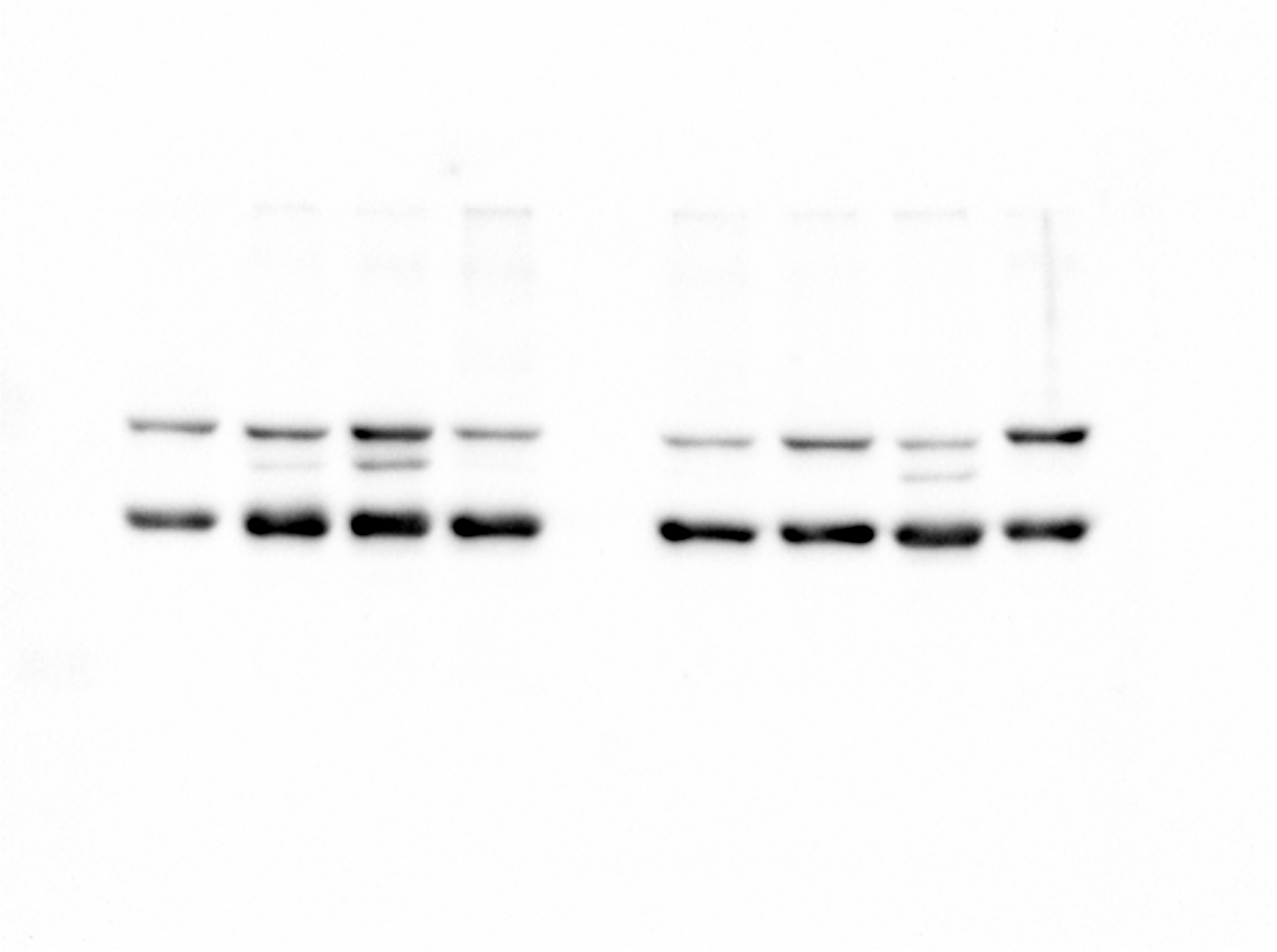

Supplement: Supplementary file 2 [file Image2.tif]

## Slide 1
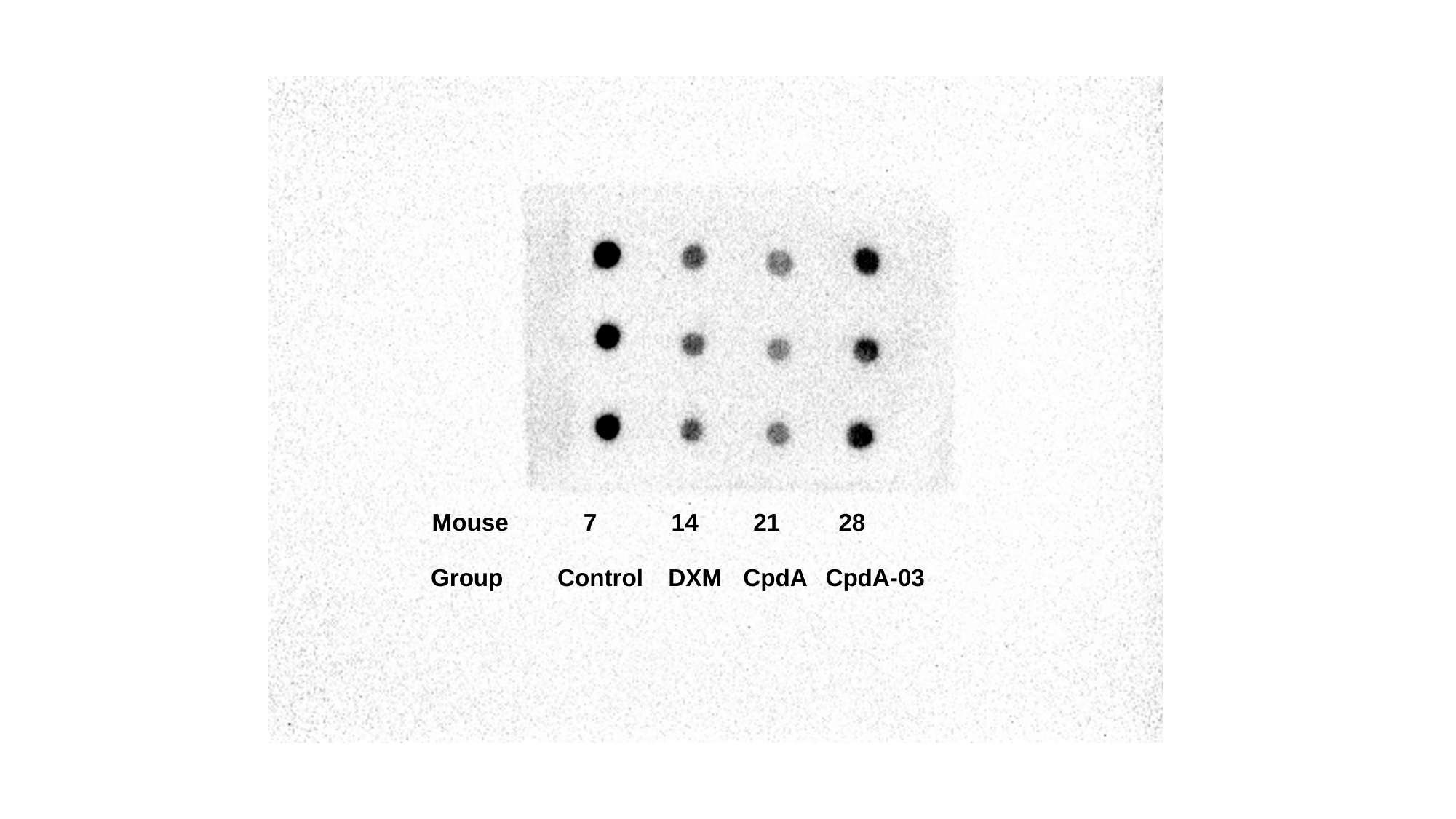

Mouse
7
14
21
28
Group
Control
DXM
CpdA
CpdA-03

Supplement: Supplementary file 3 [file Presentation1.pptx]

## Slide 1
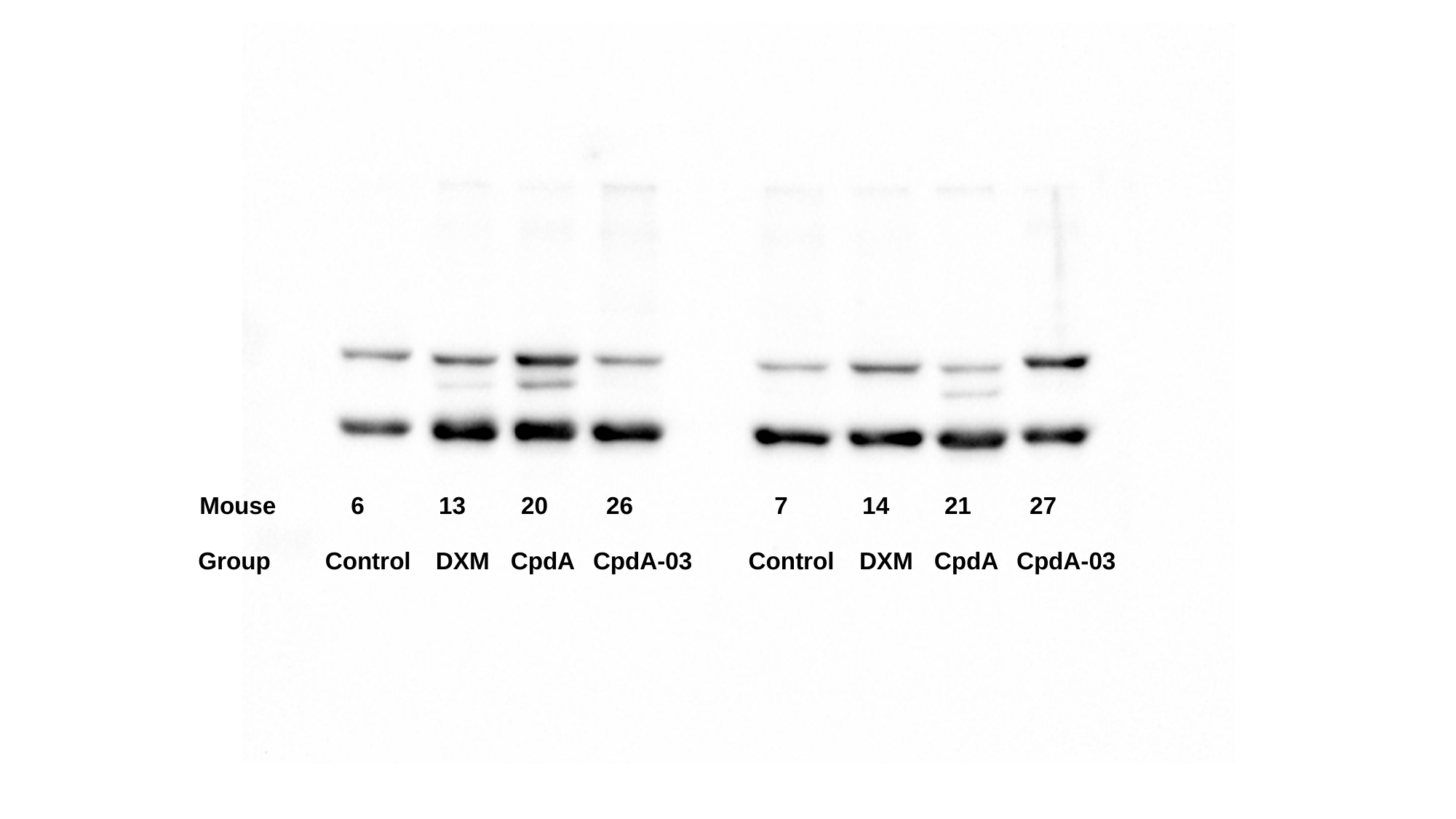

7
14
21
27
Mouse
6
13
20
26
Control
DXM
CpdA
CpdA-03
Group
Control
DXM
CpdA
CpdA-03

Supplement: Supplementary file 4 [file Presentation2.pptx]
